# Early retinal changes in type 2 diabetes detected by texture-based OCT analysis: potential approach for subclinical diabetic retinopathy diagnosis

**DOI:** 10.1186/s40662-025-00451-3

**Published:** 2025-09-03

**Authors:** Sara Oliveira, Pedro Guimarães, Ângelo Roque-Rosado, Elisa Julião Campos, Pedro Serranho, Paulo Matafome, Rui Bernardes, António Francisco Ambrósio

**Affiliations:** 1 Faculty of Medicine, Coimbra Institute for Clinical and Biomedical Research (iCBR), University of Coimbra, Coimbra, Portugal; 2 Center for Innovative Biomedicine and Biotechnology (CiBB), University of Coimbra, Coimbra, Portugal; 3 Clinical Academic Center of Coimbra (CACC), Coimbra, Portugal; 4 Coimbra Institute for Biomedical Imaging and Translational Research (CIBIT), Institute for Nuclear Sciences Applied to Health (ICNAS), University of Coimbra, Coimbra, Portugal; 5 Faculty of Medicine, University of Coimbra, Coimbra, Portugal; 6 Department of Chemical Engineering, Chemical Engineering and Renewable Resources for Sustainability (CEReS), University of Coimbra, Coimbra, Portugal; 7 Center for Neuroscience and Cell Biology (CNC), University of Coimbra, Coimbra, Portugal; 8 Department of Sciences and Technology, Universidade Aberta, Lisbon, Portugal; 9 Faculty of Medicine, Institute of Physiology, University of Coimbra, Coimbra, Portugal; 10 H&TRC—Health & Technology Research Center, Polytechnic University of Coimbra, Coimbra Health School (ESTeSC), Coimbra, Portugal

**Keywords:** Diabetic retinopathy, Early biomarkers, Optical coherence tomography, Texture analysis, Type 2 diabetes

## Abstract

**Background:**

Diabetic retinopathy (DR) is often diagnosed many years after diabetes onset, highlighting the need for early diagnosis. The current study aimed to assess whether texture analysis of computed optical coherence tomography (OCT) retinal images can identify (very) early retinal changes. We previously reported retinal texture changes in a type 1 diabetes animal model. This study extends this approach to a type 2 diabetes model exhibiting subtler, more gradually developing retinal alterations to further explore its potential for detecting texture changes when DR-related retinal alterations are minor, strengthening its promising value.

**Methods:**

OCT scans and electroretinograms were acquired at baseline and 4, 8, and 12 weeks after initiating the diabetes induction protocol. Automated OCT segmentation, retinal thickness computation, and texture analysis were performed. Blood-retinal barrier permeability, glial reactivity, neuroinflammation, and nitrosative stress were assessed.

**Results:**

Retinal texture was affected in the inner plexiform layer and inner/outer photoreceptor segments. At weeks 8 and 12, autocorrelation, cluster prominence, correlation, homogeneity, information measure of correlation II, inverse difference moment normalised, inverse difference normalised, and sum average texture metrics significantly increased/decreased. Importantly, seven of these metrics were also altered in our previous study with type 1 diabetic animals. Type 2 diabetic retinas presented subtle thinning and impaired function, along with a slight reduction in tight junction proteins immunoreactivity, without affecting the blood-retinal barrier.

**Conclusions:**

The findings from this study indicate that texture analysis can identify subtle retinal changes during early, clinically silent stages of disease, when biological alterations remain minimal. This highlights its potential utility for the early diagnosis of diabetic retinopathy, though further clinical validation is needed.

**Supplementary Information:**

The online version contains supplementary material available at 10.1186/s40662-025-00451-3.

## Background

The most prevalent cause of blindness in working-age adults is diabetic retinopathy (DR), a global epidemic with high incidence rates, affecting more than 130 million people worldwide [1–3]. Evidence gathered in recent decades has demonstrated the significant role of neurodegeneration in the early stages of the disease. Accordingly, DR is now widely accepted as a neurovascular complication, affecting both retinal microvasculature and neural cells, accompanied by chronic low-grade neuroinflammation [4–7]. Under diabetic conditions, hyperglycaemia-induced oxidative stress and inflammation contribute to the disruption of tight junctions, leading to the impairment of the blood-retinal barrier [8, 9].

Despite significant advancements over the past two decades, and the development of several treatment options, patients with DR are often undertreated or do not receive timely intervention until later stages of the disease. In addition, there is a large gap between the diagnosis of diabetes mellitus and the detection of the first clinical signs of DR [10]. During this period, the retina undergoes irreversible (molecular and cellular) changes that are undetectable with the clinical resources currently available in clinical practice [11–14], including (ultra-widefield) retinal photography, (ultra-widefield) fluorescein angiography, optical coherence tomography (OCT), and OCT angiography. Therefore, finding novel strategies for the detection of subclinical, “silent” stages of DR may be important for improving the disease detection and management. This enables timely examination and personalised treatment, thereby contributing to reducing the incidence of visual impairment or blindness.

OCT, which provides non-invasive, high-resolution cross-sectional images of the retina, is commonly used for the diagnosis of retinal diseases [15, 16]. In current clinical practice, OCT is used essentially to assess retinal structure and thickness; however, several research groups [17–22], including our group [23–29], have shown that OCT images convey more in-depth information about the retina’s status. Our team identified significant differences in retinal texture between a transgenic mouse model of Alzheimer’s disease and the respective control mice, as well as differences along the disease’s progression [30]. In clinical studies, this approach has also yielded promising results, allowing us to discriminate between Alzheimer’s disease, Parkinson’s disease, and healthy controls [31]. More recently, we conducted a longitudinal study using an animal model of streptozotocin (STZ)-induced type 1 diabetes with severe hyperglycaemia [32]. In this study, a similar texture-based methodology was applied for the first time in the context of diabetes to evaluate the potential of texture analysis in assessing early changes in the diabetic retina. Early after diabetes onset, we identified significant differences between diabetic and control animals in the following retinal texture metrics across all retinal layers: autocorrelation, correlation, homogeneity, information measure of correlation II (IMCII), inverse difference moment normalised (IDN), inverse difference normalised (INN), and sum average. These texture changes were concomitant with structural, functional, molecular, and cellular retinal alterations.

We previously demonstrated the ability to detect retinal texture changes in an animal model of type 1 diabetes, which exhibits severe hyperglycaemia and early development of molecular and cellular retinal alterations. Thus, in this study, the same methodology was applied using an animal model of type 2 diabetes. Here, retinal changes are more subtle and progress more gradually. Accordingly, the present study sought to investigate whether retinal texture is also altered when milder diabetes-induced changes occur in the retina. By exploring texture changes in an animal model with subtler retinal changes, this study explores the potential of our texture-based approach for detecting (very) early retinal alterations, thereby further underscoring the relevance of this strategy and validating its promising value for the early detection of DR, even at “silent” stages. As a proof of concept, this longitudinal study used Wistar Han rats (male and female rats) induced by a high-fat diet (HFD) and a low-dose intraperitoneal (IP) injection of STZ (35 mg/kg). To assess whether potential retinal texture changes occur concomitantly, or not, with DR-related biological alterations in the retina, OCT and electroretinography (ERG) acquisitions were performed, along with the evaluation of molecular, cellular, and vascular parameters. The grey-level co-occurrence matrix (GLCM), a statistical method that provides a statistical distribution of grey levels in an image, was used to extract texture metrics from the OCT-derived images of rat retinas.

Animal models are fundamental for providing critical insights into DR pathophysiology, diagnosis, and treatment. Although no single animal model of diabetes precisely recapitulates every aspect of human DR, studies conducted with the animal model employed in this study (HFD/STZ-induced diabetic rats) reported hyperglycaemia, obesity, retinal function abnormalities, decreased retinal thickness, increased acellular capillaries, and decreased number of pericytes [33–35]. As texture refers to a complex characterisation of the structural arrangement of image regions and does not possess a (direct) relation with a specific biological process, it is important to clarify whether the potential changes in retinal texture are concomitant, or not, with biological alterations in the retina. Therefore, performing an animal study is indispensable, allowing us to evaluate biological parameters that cannot be assessed in vivo in humans, such as the immunoreactivity of tight junction proteins and the levels of pro-inflammatory mediators in the retina. It also enables the assessment of retinal changes (very) early after diabetes onset, potentially at disease stages that would not be detectable with the diagnostic resources currently used in ophthalmological practice. Accordingly, this study hypothesises that texture analysis holds the potential for detecting retinal changes at subclinical, “silent” stages of DR, when the retina is undergoing very subtle and early (molecular and cellular) alterations.

## Methods

### Animal model and ethics

All animal procedures were performed in compliance with the European Community directive guidelines (2010/63/EU) for the use of experimental animals, transposed into Portuguese law in 2013 (Decreto-lei 113/2013). The procedures were approved by the Animal Welfare Committee of the Coimbra Institute for Clinical and Biomedical Research (iCBR), Faculty of Medicine, University of Coimbra (ORBEA 02/2021), and received further approval from the Direção Geral de Alimentação e Veterinária (DGAV – approval no. 0421/000/000/2021). Additionally, the animal experimentation was conducted in accordance with the Association for Research in Vision and Ophthalmology (ARVO) Statement for the Use of Animals in Ophthalmic and Vision Research.

**Fig. 1:**
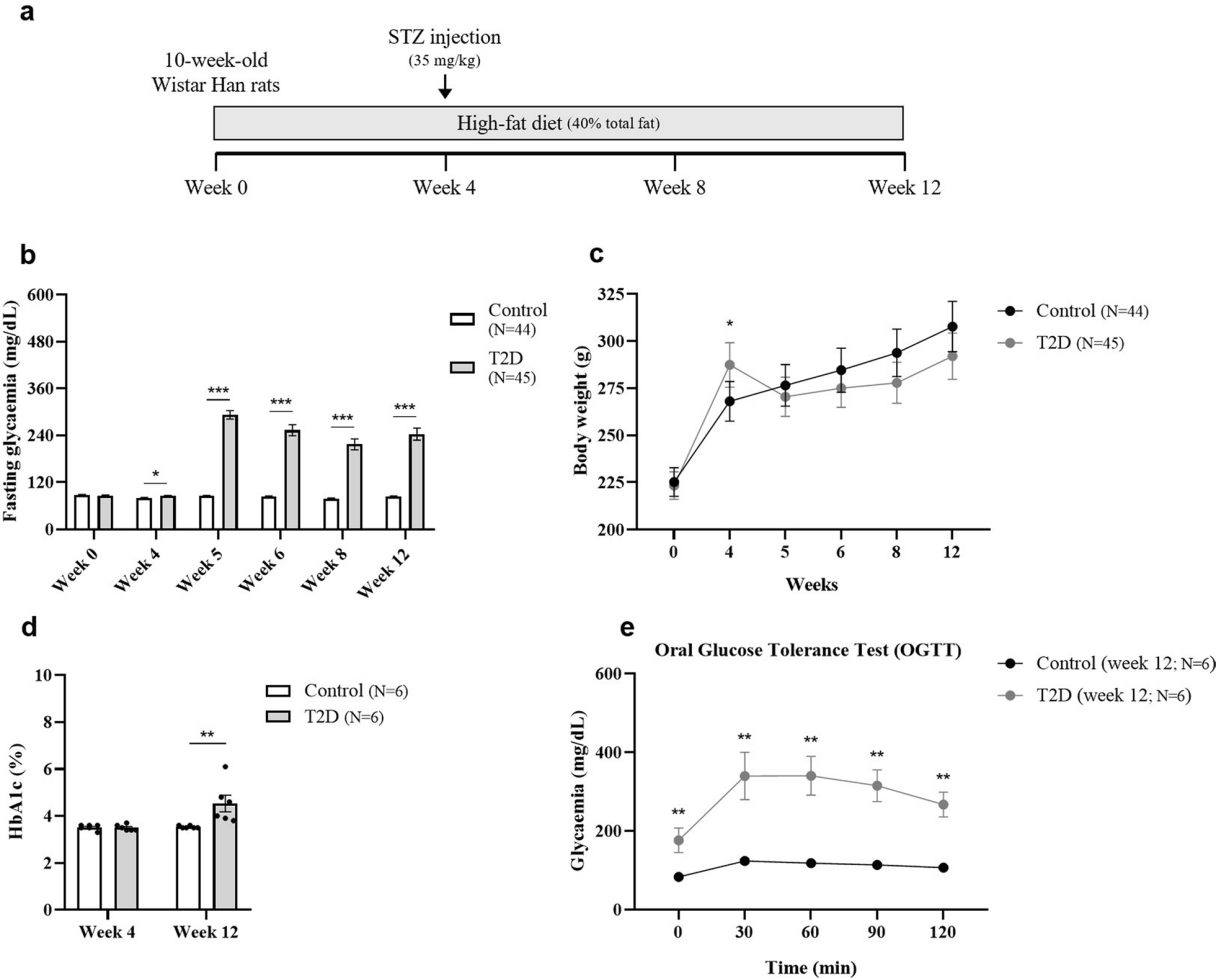
Impact of diabetes on body weight and metabolic parameters. (**a**) Protocol of type 2 diabetes induction. Animals were fed a high-fat diet (HFD) for 12 weeks, combined with a single low-dose STZ injection (35 mg/kg, IP) in the fourth week after starting the diet. (**b**) Fasting glycaemia (6 h fasting), (**c**) body weight, and (**d**) HbA1c were monitored in diabetic Wistar Han rats (T2D group) and age-matched controls (Control group). (**e**) The OGTT (1.8 g/kg glucose) was performed after 6 h of fasting (only the results obtained after 12 weeks on HFD are presented). Results are presented as mean ± SEM. Statistical analysis was performed using the Mann–Whitney test. *P ≤ 0.05, **P < 0.01, ***P < 0.001, versus the age-matched Control group. STZ, streptozotocin; IP, intraperitoneal; HbA1c, glycated haemoglobin; T2D, type 2 diabetes; OGTT, oral glucose tolerance test; SEM, standard error of the mean

Male and female (10-week-old) Wistar Han rats (Charles River Laboratories, Lyon, France) were housed in certified facilities with a temperature- and humidity-controlled environment, maintained under a 12 h:12 h light–dark cycle, and with ad libitum access to food and water. The animals were randomly divided into the control and diabetic (T2D) groups. Type 2 diabetes was induced by a HFD containing approximately 40% total fat (Special Diet HF231—PF4486; Mucedola srl, Settimo Milanese, Italy) for 12 weeks, in combination with a single low-dose STZ injection (35 mg/kg in 10 mM sodium citrate buffer, pH 4.5; IP; after 6 h fasting) in the fourth week after starting the diet (Fig. 1a). Fasting glycaemia (Fig. 1b) and body weight (Fig. 1c) were monitored at the beginning of the study, i.e., just before starting the diet (week 0), and after 4, 5, 6, 8, and 12 weeks on HFD. Glycated haemoglobin (HbA1c) was measured at weeks 4 and 12 (Fig. 1d), and the oral glucose tolerance test (OGTT; 1.8 mg/kg glucose) was performed at weeks 0 and 12 (the results for week 12 are presented in Fig. 1e).

### OCT data acquisition

Animals were anesthetised with an IP injection of ketamine (30 mg/kg; Nimatek, Dechra, Northwich, UK) and medetomidine (1 mg/kg; Sedator, Dechra, Northwich, UK) in 0.1 mL saline solution, followed by corneal anaesthesia with oxybuprocaine (4 mg/mL; Anestocil, Edol, Lisbon, Portugal), and full pupil dilation using a topical drop of tropicamide (10 mg/mL; Tropicil Top, Edol, Lisbon, Portugal). To keep the corneal surface moisturised, carmellose sodium (4 mg/0.4 mL; Celluvisc, Allergan, Dublin, Ireland) lubricating eye drops were regularly applied. The retinas from the right eye of control (*N* = 44) and diabetic (*N* = 45) animals were imaged using a Micron IV OCT System (Phoenix Technology Group, Pleasanton, CA, USA) at the baseline (week 0) and after 4, 8, and 12 weeks on HFD. Retinal OCT volumes were acquired consisting of 512 B-scans each (composed of 512 A-scans of 1024 pixels in length), imaged at a predefined retinal region (located above the optic disc, maintaining a horizontal alignment with its centre). The B-scans were saved as non-compressed TIFF image files. All OCT data acquisitions were performed by the same operator. In the molecular and cellular studies, multiple parameters were assessed using retinal samples from the same animals.

### Automatic retinal segmentation and retinal thickness measurement

The retinal layer segmentation was achieved using a fully convolutional neural network, following a ResNet architecture, as previously described [32, 36, 37]. A total of six distinct structures (layers and layer aggregates) were segmented: the nerve fiber layer and ganglion cell layer complex (NFL-GCL), the inner plexiform layer (IPL), the inner nuclear layer (INL), the outer plexiform layer (OPL), the outer nuclear layer (ONL), and the inner/outer photoreceptor segments (IS/OS). Volumetric segmentation was determined by combining the 512 segmented B-scans. An expert (author S.O.) evaluated the quality of the segmentation in a masked fashion for both experimental groups. After obtaining the volumetric segmented OCT data, retinal thickness maps were computed for each of the six retinal layers, and the thickness of each retinal layer/layer conjugate, as well as the thickness of the entire retina, were calculated as the distance between the respective segmented boundaries, as reported in our previous work [32].

### Texture analysis

For each layer, projection mean-value fundus (MVF) images [25] were computed from volume OCT data, as previously described [32]. MVF images consist of 2-dimensional (2D) images in which each pixel’s value is the average of the values of each A-scan between two retinal layer interfaces defining the layer. These allow the projection of the 3-dimensional (3D) OCT data onto a 2D plane, which mimics a fundus photograph capturing only the layer of interest. Texture-related features were extracted from the MVF images using the GLCM statistical method, as described previously [32]. The key concept of GLCM lies in analysing the probability of finding a transition from a grey-level A to a grey-level B along an established direction and distance in the image. By computing all possible different transitions at a pre-defined pixel distance and direction, GLCM provides texture information of the image being analysed. Twenty features per layer were computed, namely: the inverse difference moment/energy, contrast/inertia, correlation, angular second moment/uniformity/homogeneity, sum average, sum of squares, sum variance, sum entropy, difference variance, difference entropy, information measure of correlation I (IMCI), IMCII, and entropy, as described by Haralick et al. [38]; autocorrelation and maximum probability, as described by Haralick et al. [39]; cluster prominence and cluster shade, as described by Conners et al. [40]; INN and IDN, as described by Clausi et al. [41]; and dissimilarity, as described by Soh et al. [42].

### ERG

Animals were anesthetised as described above, and electroretinograms were recorded at baseline (week 0) and after 4, 8, and 12 weeks on HFD, following the protocol from our previous study [32]. In brief, after a dark adaptation overnight, retinal ganglion cell (RGC) function was registered by stimulating both eyes with a 0.000095 cd·s/m^2^ light stimulus, following the scotopic threshold response (STR) protocol. The scotopic luminance response was also recorded under scotopic conditions by stimulation with light flashes ranging from 0.0095 to 9.49 cd·s/m^2^, inducing rod activation (a-wave), followed by the subsequent activation of the downstream retinal cells [oscillatory potentials (OPs) and b-wave]. Then, after light adaptation to a 25 cd/m^2^ white background for 15 min, the photopic luminance response was recorded in response to a 0.0095 to 9.49 cd·s/m^2^ light stimulus. To obtain a more isolated response from cones, the photopic flicker protocol was performed by stimulation with white light flashes (0.95, 3.00, and 9.49 cd·s/m^2^) delivered 10 times at 6.33 Hz. ERGs were recorded using a RETIport System (Roland Consult GmbH, Brandenburg, Germany), and the light stimulation was performed using a Ganzfeld stimulator (Roland Consult GmbH, Brandenburg, Germany), with the data being extracted from the RETIport software (Roland Consult GmbH, Brandenburg, Germany).

Following OCT and ERG recordings, atipamezole (1 mg/kg; Revazol, Dechra, Northwich, UK) was injected intraperitoneally to reverse medetomidine sedation.

### Immunofluorescence

#### Retinal wholemounts

Animals were anesthetised (2.5% isoflurane in O_2_; IsoFLO, Ecuphar, Oostkamp, Belgium) and euthanised by cervical dislocation. The retinas were then dissected, and the retinal wholemounts were prepared following the procedure previously reported by our group [32]. Briefly, after fixation with 4% paraformaldehyde (PFA) for 10 min, the retinal wholemounts were blocked for 1 h with 5% bovine serum albumin (BSA) and incubated for 72 h with antibodies against claudin-5, occludin, and zonula occludens-1 (ZO-1; Table 1). After washing, the retinas were incubated with the corresponding secondary antibodies (Table 1) for 24 h, followed by incubation with 1:1000 DAPI (Thermo Fisher Scientific, Waltham, MA, USA), and then mounted with the vitreous side up for visualisation under the LSM 710 Meta confocal laser scanning microscope (Zeiss, Jena, Germany) with a 20 × objective (Plan Achromat 20 × /0.8 M27).

**Table 1:** List of antibodies used for immunofluorescence of retinal wholemounts

Antibody	Dilution	Company	Catalogue number
Mouse anti-occludin	1:100	Invitrogen	33–1500
Rabbit anti-claudin-5	1:100	Invitrogen	34–1600
Rabbit anti-ZO-1	1:100	Invitrogen	40–2200
AlexaFluor 488 goat anti-rabbit	1:200	Invitrogen	A-11008
AlexaFluor 568 goat anti-mouse	1:200	Invitrogen	A-11004

#### Retinal cryosections

Retinal sections (14-μm thick) were prepared as described previously [32]. Briefly, these were fixed in cold acetone for 10 min, permeabilized for 30 min, and blocked with 10% normal goat serum and 1% BSA before overnight incubation with primary antibodies against Iba1, OX-6/MHC II, glial fibrillary acidic protein (GFAP), vimentin, and nitrotyrosine (at 4 °C; Table 2). Sections were then washed and incubated with the secondary antibodies (Table 2) and 1:5000 DAPI for 1 h at room temperature. For image analysis, the images were captured with a fluorescence microscope (Axio Observer.Z1, Zeiss, Jena, Germany) using a 20 × objective (Plan Achromat 20 × /0.8 M27). Representative Z-stack images were acquired with a 20 × objective (Plan Achromat 20 × /0.8 M27) on a confocal microscope (Zeiss LSM 710, Zeiss, Jena, Germany).

**Table 2:** List of antibodies used for immunofluorescence of retinal sections

Antibody	Dilution	Company	Catalogue number
Rabbit anti-nitrotyrosine	1:100	Millipore	06-284
Rabbit anti-Iba1	1:1000	Wako	019-19741
Mouse anti-MHC Class II (OX-6)	1:200	Bio-Rad	MCA46A647
Chicken anti-GFAP	1:500	Millipore	AB5541
Rabbit anti-vimentin	1:500	Abcam	ab92547
AlexaFluor 488 goat anti-rabbit	1:200	Invitrogen	A-11008
AlexaFluor 568 goat anti-chicken	1:200	Invitrogen	A-11041
AlexaFluor 568 goat anti-mouse	1:200	Invitrogen	A-11004

### Evans blue assay

Blood-retinal barrier permeability was qualitatively assessed using the Evans blue assay, following our group’s previously published protocol [32]. Animals were anesthetised with an IP injection of ketamine (75 mg/kg; Nimatek, Dechra, Northwich, UK) and medetomidine (1 mg/kg; Sedator, Dechra, Northwich, UK). After filtration (pore size, 0.2 μm), Evans blue (100 mg/kg in PBS; Sigma-Aldrich, St. Louis, MO, USA) was injected via the tail vein, and the animals were kept on a warm pad for 2 h. The eyes were enucleated and fixed with 2% PFA for 2 h, after which the retinas were dissected and mounted with the vitreous side up for visualisation under the LSM 710 Meta confocal laser scanning microscope (Zeiss, Jena, Germany) with a 20 × objective (Plan Achromat 20 × /0.8 M27).

### Western blotting

Retinal lysates were prepared, and Western blot was performed as described previously [32]. In brief, equal amounts of protein (30 µg) were loaded onto 6%–12% polyacrylamide gels, separated through sodium dodecyl sulphate–polyacrylamide gel electrophoresis (SDS-PAGE), and transferred to polyvinylidene fluoride membranes (Millipore, Billerica, MA, USA). After blocking for 2 h at room temperature, membranes were incubated overnight with claudin-5, occludin, ZO-1, interleukin-1 beta (IL-1β), and tumour necrosis factor (TNF) primary antibodies (Table 3), followed by 2 h incubation with the corresponding secondary antibodies (Table 3). Immunoblots were imaged on the ImageQuant™ LAS 500 (GE Healthcare, Chicago, IL, USA), and band intensity was quantified using ImageQuant 5.0 software (Molecular Dynamics, Sunnyvale, CA, USA).

**Table 3:** List of antibodies used for Western blotting

Antibody	Dilution	Company	Catalogue number
Rabbit anti-occludin	1:200	Invitrogen	71-1500
Rabbit anti-claudin-5	1:200	Invitrogen	34-1600
Rabbit anti-ZO-1	1:200	Invitrogen	40-2200
Rabbit anti-IL-1β	1:200	Abcam	ab9722
Rabbit anti-TNF	1:200	Abcam	ab66579
Goat anti-calnexin	1:1000	Sicgen	AB0041-500
HRP goat anti-rabbit	1:10,000	Bio-Rad	1706515
HRP rabbit anti-goat	1:10,000	Invitrogen	61-1620

### Enzyme-linked immunosorbent assay

For tissue preparation, one retina from each eye was homogenised in lysis buffer [20 mM imidazole HCl, 100 mM KCl, 1 mM MgCl_2_, 1% (vol./vol.) Triton X-100, 1 mM EGTA, 1 mM EDTA, 10 mM NaF, and 1 mM Na_3_VO_4_ (activated), supplemented with complete mini protease inhibitor cocktail tablets]. Then, following the manufacturer’s instructions, the protein levels of IL-1β and TNF were assessed in retinal homogenates using the rat IL-1β and TNF enzyme-linked immunosorbent assay (ELISA) kits (#RAB0478 and #RAB0480; Sigma-Aldrich, St. Louis, MO, USA), respectively.

### Statistical analysis

ANCOVA tests were performed to assess differences between groups and each individual time point of the study (after 4, 8, and 12 weeks on HFD) after adjusting for the baseline (week 0), which was used as a quantitative covariate. Additionally, the results from retinal thickness, metabolic parameters, Western blot, and immunofluorescence were analysed using the Student’s t-test, or the Mann–Whitney test, if data were not normally distributed, which was assessed using the Shapiro–Wilk test. SPSS software (version 27; IBM Corp., Armonk, NY, USA) was used to perform the statistical analysis, where we considered a statistical significance of 5%. Results are presented as mean ± standard error of the mean (SEM), except for the graphs of the retinal texture, in which the results are expressed by box plots showing the median (horizontal line), 25th and 75th percentiles (box), and minimum and maximum (error bars). The results of the retinal thickness were calculated as the percentage of the baseline and are presented as mean ± SEM.

## Results

### Type 2 diabetes triggers early texture changes in specific retinal layers

**Fig. 2:**
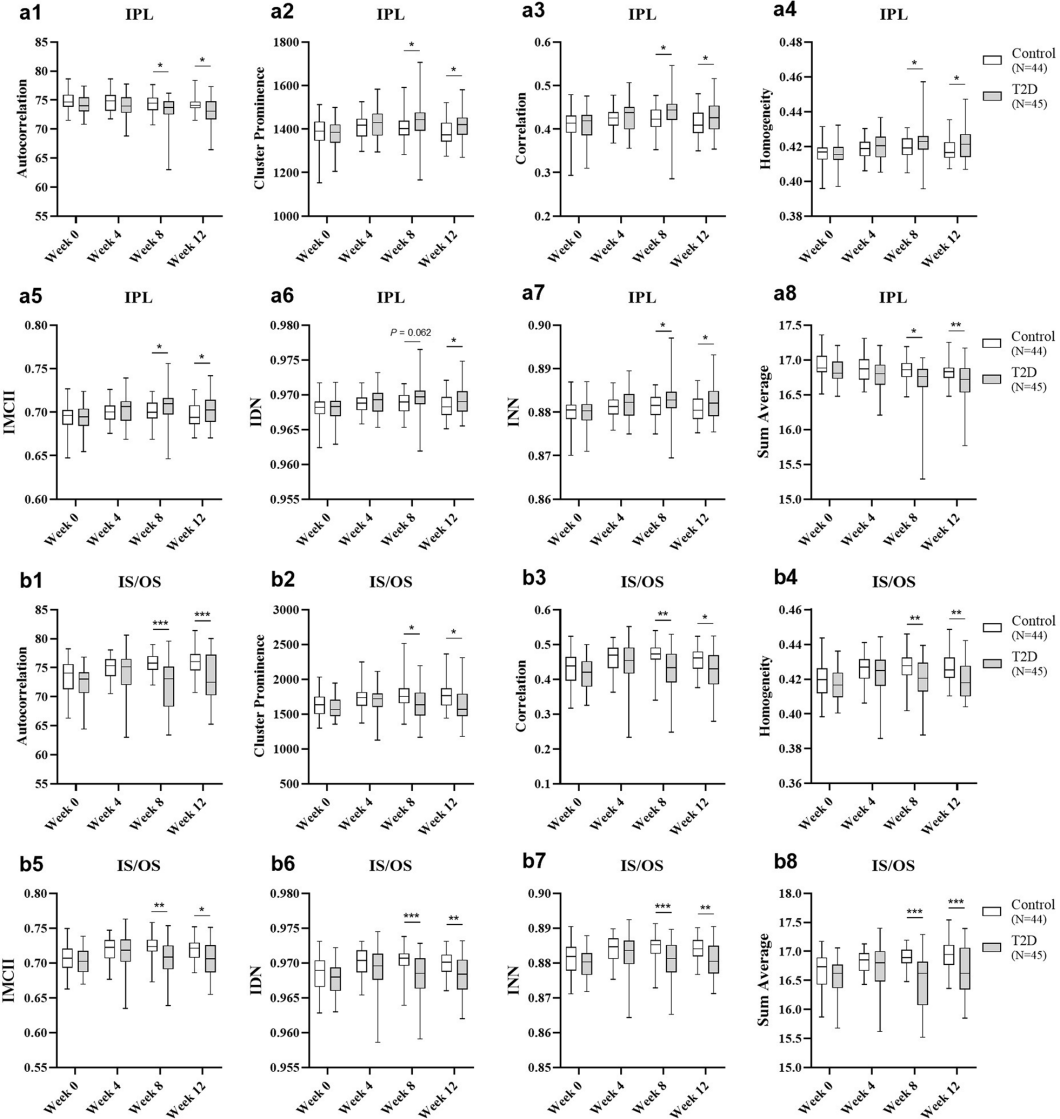
Diabetes triggers (very) early texture changes extracted from retinal OCT images. Diabetic Wistar Han rats (induced by a 12-week HFD with STZ injection at week 4; 35 mg/kg, IP; T2D group; *N* = 45), presented changes in retinal texture, compared with age-matched controls (Control group; *N* = 44), in the following GLCM-based textural parameters in the inner plexiform layer (IPL) and inner/outer photoreceptor segments (IS/OS), respectively: (**a1**, **b1**) autocorrelation; (**a2**, **b2**) cluster prominence; (**a3**, **b3**) correlation; (**a4**, **b4**) homogeneity; (**a5**, **b5**) information measure of correlation II (IMCII); (**a6**, **b6**) inverse difference moment normalised (IDN); (**a7**, **b7**) inverse difference normalised (INN); (**a8**, **b8**) sum average. The results are expressed by box plots showing the median (horizontal line), 25th and 75th percentiles (box), and minimum and maximum (error bars) for the IPL and IS/OS in each texture parameter. Statistical analysis was performed using the ANCOVA test, considering the baseline values (week 0) as a quantitative covariate. *P ≤ 0.05, ** P < 0.01, *** P < 0.001, versus the age-matched Control group. OCT, optical coherence tomography; STZ, streptozotocin; IP, intraperitoneal; T2D, type 2 diabetes; GLCM, grey-level co-occurrence matrix; HFD, high-fat diet

We previously identified significant texture changes in retinal OCT images obtained from an animal model of type 1 diabetes, early after diabetes onset, suggesting that texture analysis offers a promising approach for the early detection of DR [32]. Accordingly, the main goal of the present work is to evaluate the potential of this texture analysis-based approach in HFD/STZ-induced type 2 diabetic animals, in which molecular and cellular changes are subtler and progress more slowly. It is important to highlight that animals on HFD for 8 and 12 weeks were injected with STZ at week 4 (Fig. 1a). After 8 and 12 weeks on HFD, significant changes were found in several GLCM-based texture metrics in specific retinal layers, namely in the IPL and IS/OS, compared to control animals. More specifically, the IPL presented a significant decrease in the texture metrics autocorrelation (Fig. 2a1) and sum average (Fig. 2a8); conversely, there was an increase in the texture metrics cluster prominence (Fig. 2a2), correlation (Fig. 2a3), homogeneity (Fig. 2a4), IMCII (Fig. 2a5), and INN (Fig. 2a7), compared to control animals. A significant increase in the IDN texture parameter was also observed, but only at week 12 (Fig. 2a6). The same texture parameters were affected in the retinal layer IS/OS (Fig. 2b1–8). However, in contrast to the IPL, all these parameters significantly decreased after 8 and 12 weeks on HFD, compared with controls. After 4 weeks on HFD, no changes were observed in any of these texture metrics. The mean values ± SEM for each parameter assessed and the corresponding significance *P* values are provided in Supplementary Table S1.

### Type 2 diabetes significantly impacts retinal thickness

**Fig. 3:**
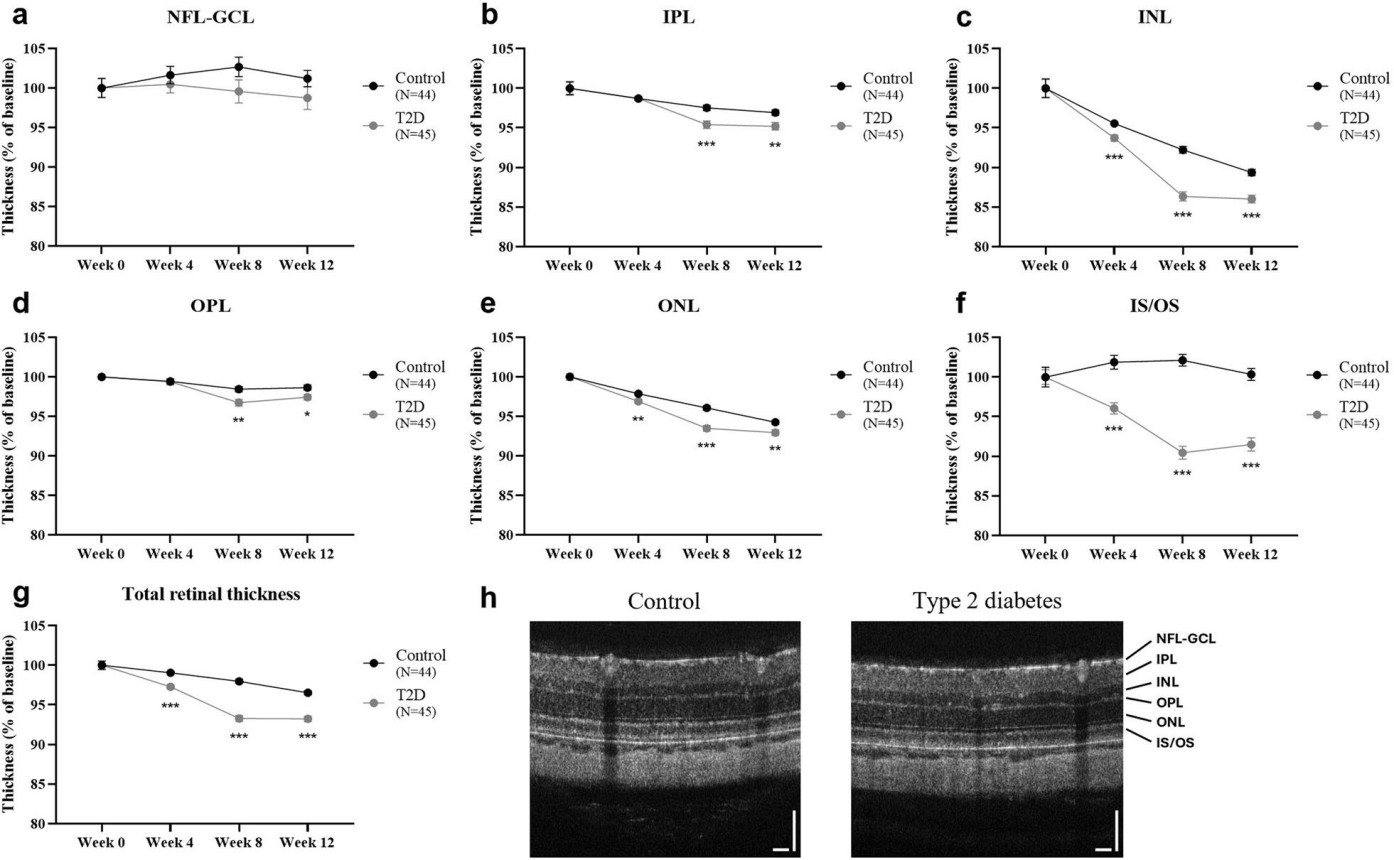
Diabetes induces retinal thinning. Diabetic Wistar Han rats (induced by a 12-week HFD with STZ injection at week 4; 35 mg/kg, IP; T2D group; *N* = 45) presented a decrease in retinal thickness compared to age-matched controls (Control group; *N* = 44). The retinal thickness of control and diabetic rats was assessed in the following retinal layers: (**a**) nerve fibre layer and ganglion cell layer complex (NFL-GCL); (**b**) inner plexiform layer (IPL); (**c**) inner nuclear layer (INL); (**d**) outer plexiform layer (OPL); (**e**) outer nuclear layer (ONL); (**f**) inner/outer photoreceptor segments (IS/OS). (**g**) Total retinal thickness was calculated as the sum of the six retinal layers. (**h**) Representative OCT images for Control and T2D groups after 12 weeks on HFD. Scale bar: 100 μm. Results were calculated as the percentage of the baseline and are presented as mean ± SEM. Statistical analysis was performed using the Student’s *t*-test, or the Mann–Whitney test, if data were not normally distributed. *P ≤ 0.05, ** P < 0.01, *** P < 0.001, versus the age-matched Control group. HFD, high-fat diet; STZ, streptozotocin; IP, intraperitoneal; T2D, type 2 diabetes; SEM, standard error of the mean

The effect of diabetes on retinal thickness was assessed on the segmented retinal layers (NFL-GCL, IPL, INL, OPL, ONL, and IS/OS) as well as on the total retina (Fig. 3a–h). Almost all retinal layers were significantly thinner in diabetic animals after 8 and 12 weeks on HFD, compared to controls, as follows: IPL [Control: *N* = 44, 97.5% ± 0.4% and 96.9% ± 0.3% vs. T2D: *N* = 45, 95.4% ± 0.5% (*P* < 0.001) and 95.2% ± 0.5% (*P* = 0.004) at weeks 8 and 12, respectively; Fig. 3b], INL (Control: *N* = 44, 92.2% ± 0.4% and 89.4% ± 0.4% vs. T2D: *N* = 45, 86.3% ± 0.6% and 86.0% ± 0.5% at weeks 8 and 12, respectively, *P* < 0.001; Fig. 3c), OPL [Control: *N* = 44, 98.4% ± 0.3% and 98.6% ± 0.3% vs. T2D: *N* = 45, 96.7% ± 0.4% (*P* = 0.002) and 97.4% ± 0.4% (*P* = 0.013) at weeks 8 and 12, respectively; Fig. 3d], ONL [Control: *N* = 44, 96.1% ± 0.3% and 94.3% ± 0.3% vs. T2D: *N* = 45, 93.5% ± 0.4% (*P* < 0.001) and 93.0% ± 0.3% (*P* = 0.008) at weeks 8 and 12, respectively; Fig. 3e], and IS/OS (Control: *N* = 44, 102.1% ± 0.7% and 100.3% ± 0.8% vs. T2D: *N* = 45, 90.5% ± 0.8% and 91.5% ± 0.8% at weeks 8 and 12, respectively, *P* < 0.001; Fig. 3f). Retinal thinning was also observed in the T2D group after 4 weeks on HFD, namely in the INL and ONL [Control: *N* = 44, 95.5% ± 0.4% and 97.9% ± 0.2% vs. T2D: *N* = 45, 93.7% ± 0.4% (*P* = 0.001) and 96.9% ± 0.3% (*P* = 0.010), respectively] and in the photoreceptor segments (Control: *N* = 44, 101.9% ± 0.9% vs. T2D: *N* = 45, 96.1% ± 0.7%, *P* < 0.001). As a result, a significant decrease in the total retinal thickness (Fig. 3g) was observed in diabetic rats after 4, 8, and 12 weeks on HFD (Control: *N* = 44, 99.1% ± 0.3%, 98.0% ± 0.3%, and 96.5% ± 0.3% vs. T2D: *N* = 45, 97.3 ± 0.3%, 93.3 ± 0.4%, and 93.2 ± 0.3% at weeks 4, 8, and 12, respectively, *P* < 0.001), compared to age-matched controls.

### Type 2 diabetes increases the latency of the OPs under scotopic conditions

**Fig. 4:**
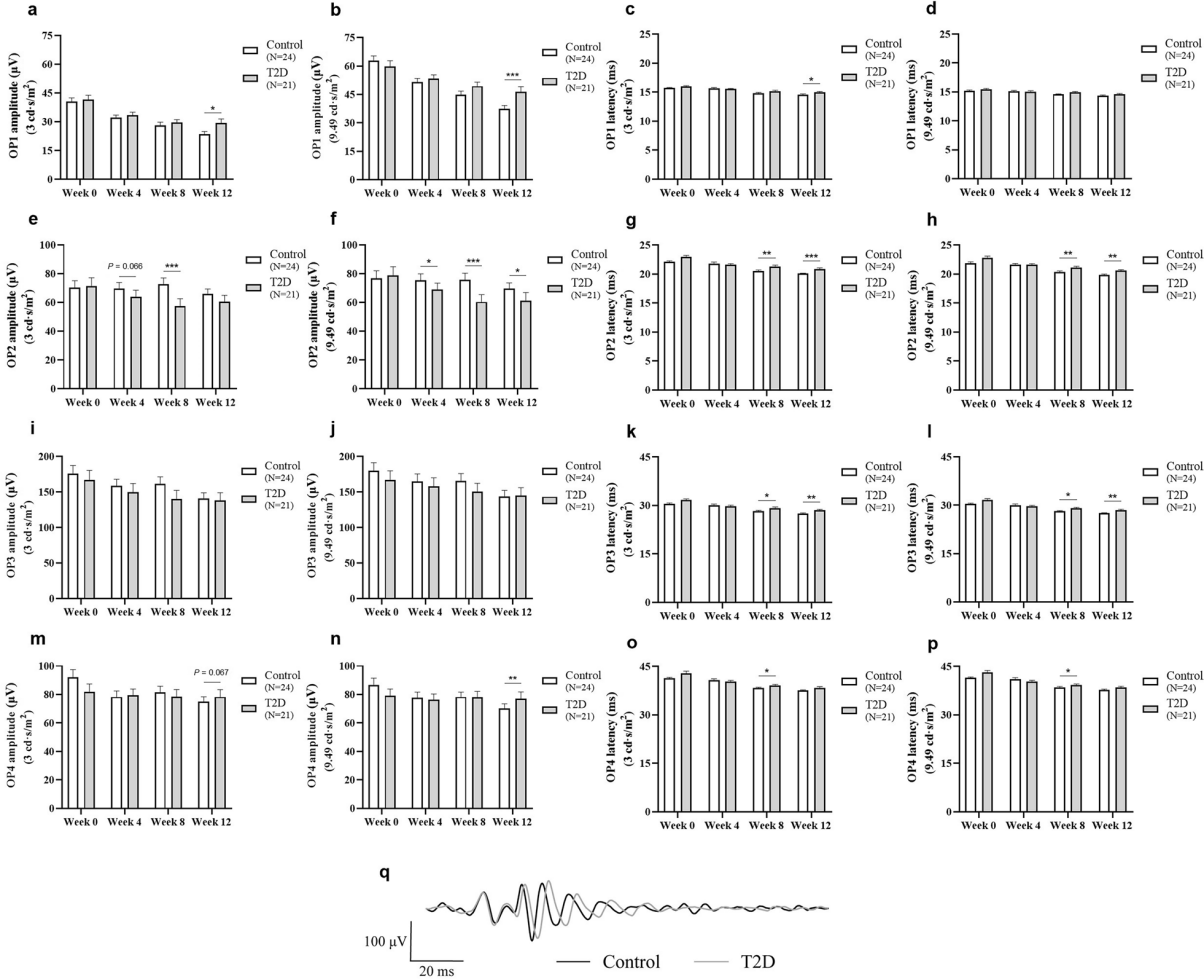
Impact of diabetes on oscillatory potentials (OPs). The response of the scotopic OPs was delayed in diabetic Wistar Han rats (induced by a 12-week HFD with STZ injection at week 4; 35 mg/kg, IP; T2D group; *N* = 21), compared to age-matched controls (Control group; *N* = 24). OP1, OP2, OP3, and OP4 amplitudes were recorded in response to (**a**, **e**, **i**, and **m**, respectively) 3 cd·s/m^2^ and (**b**, **f**, **j**, and **n**, respectively) 9.49 cd·s/m^2^ light stimuli. Latencies of OP1, OP2, OP3, and OP4 were recorded in response to (**c**, **g**, **k**, and **o**, respectively) 3 cd·s/m^2^ and (**d**, **h**, **l**, and **p**, respectively) 9.49 cd·s/m^2^ light stimuli. (**q**) Representative traces of OPs of control and diabetic animals, recorded after 12 weeks on HFD, were obtained after applying an Off-line digital filter (low frequency cutoff of 60 Hz). Results are presented as mean ± SEM. Statistical analysis was performed using the ANCOVA test, considering the baseline values (week 0) as a quantitative covariate. *P ≤ 0.05, ** P < 0.01, *** P < 0.001, versus the age-matched Control group. HFD, high-fat diet; STZ, streptozotocin; IP, intraperitoneal; T2D, type 2 diabetes

Electroretinograms, which measure the electrical response of the retina to light flashes, were performed to investigate the effect of diabetes on the retinal physiology. At all experimental time points (after 4, 8, and 12 weeks on HFD), the retinal function of diabetic animals was mostly unaffected under both scotopic (Supplementary Figure S1) and photopic conditions (Supplementary Figure S2), compared with control animals, except for the scotopic OPs (Fig. 4a–q). In OP1, increased latencies were registered after 12 weeks on HFD, in response to a 3 cd·s/m^2^ light stimulus (Control: 14.6 ± 0.1 ms vs. T2D: 15.0 ± 0.1 ms, *P* = 0.017; Fig. 4c), whereas, in OP2 and OP3, this effect was observed earlier (after 8 weeks on HFD) in response to 3 cd·s/m^2^ [Control: *N* = 24, 20.5 ± 0.2 ms and 28.2 ± 0.3 ms vs*.* T2D: *N* = 21, 21.3 ± 0.2 ms (*P* = 0.002) and 29.2 ± 0.3 ms (*P* = 0.021) in OP2 and OP3, respectively; Fig. 4g and k] and 9.49 cd·s/m^2^ light stimuli [Control: *N* = 24, 20.4 ± 0.2 ms and 28.1 ± 0.3 ms vs*.* T2D:* N* = 21, 21.1 ± 0.2 ms (*P* = 0.009) and 29.1 ± 0.3 ms (*P* = 0.011) in OP2 and OP3, respectively; Fig. 4h and l], and remained significant until week 12. Regarding the OP4, a delayed response was observed after 8 weeks on HFD under both light stimuli analysed (3 cd·s/m^2^ and 9.49 cd·s/m^2^; Fig. 4o and p, respectively), although this effect was lost at week 12.

### Type 2 diabetes induces mild changes in tight junction protein expression without causing vascular leakage

**Fig. 5:**
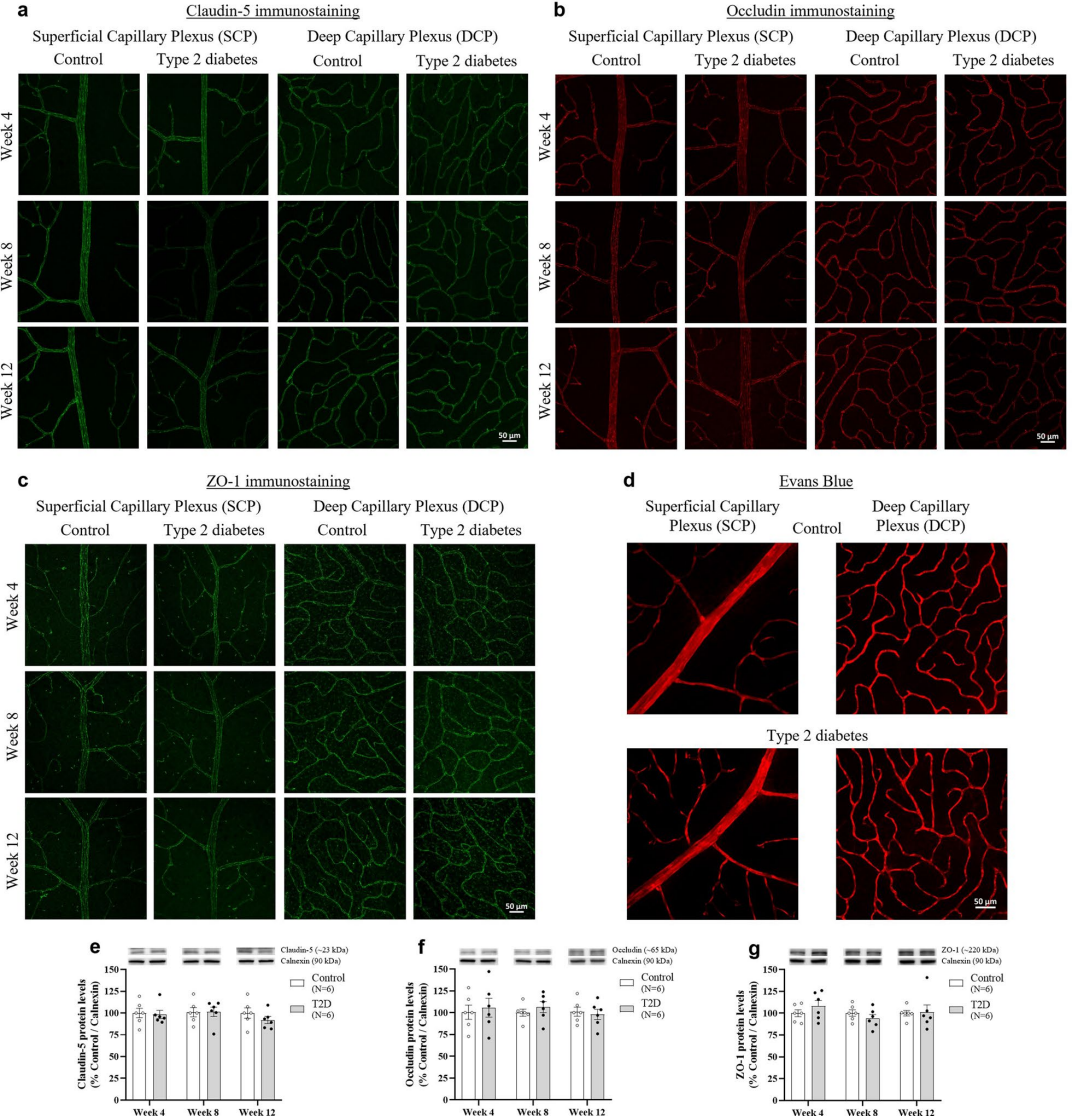
Diabetes triggers slight immunoreactive changes in tight junction proteins, without changes in retinal vascular permeability. Diabetic Wistar Han rats (induced by a 12-week HFD with STZ injection at week 4; 35 mg/kg, IP; T2D group) presented a slight decrease in claudin-5 immunoreactivity after 8 weeks on HFD, as well as in occludin and claudin-5 immunostaining at week 12, as assessed in retinal wholemounts. However, no differences were found in its protein levels in retinal extracts, assessed by Western blot, compared to age-matched controls (Control group). No vascular leakage was detected in diabetic animals, as assessed by the Evans blue assay. Representative images of retinal wholemounts immunostained for (**a**) claudin-5, (**b**) occludin, and (**c**) ZO-1. (**d**) Representative images showing Evans blue fluorescence in the retina. (**e**) Claudin-5, (**f**) occludin, and (**g**) ZO-1 protein levels assessed by Western blot, normalised to the loading control (calnexin), and expressed as percentage of the respective control. Representative images of protein immunoreactive bands are presented above the graphs, with the respective loading control (calnexin). Data are presented as mean ± SEM. Statistical analysis was performed using the Student’s *t*-test, or the Mann–Whitney test, if data were not normally distributed. HFD, high-fat diet; STZ, streptozotocin; IP, intraperitoneal; T2D, type 2 diabetes; ZO-1, zonula occludens-1

The impact of diabetes on the tight junction proteins claudin-5, occludin, and ZO-1 was assessed in retinal wholemounts and homogenates by immunofluorescence and Western blot, respectively. After 8 weeks on HFD, a qualitative analysis of the retinal wholemounts revealed a slight decrease in claudin-5 immunoreactivity in the superficial capillary plexus (SCP) in diabetic rats, which persisted until week 12, compared with controls (Fig. 5a). A subtle decrease in occludin immunoreactivity was also observed after 12 weeks on HFD, both in the SCP and in the deep capillary plexus (Fig. 5b). Regarding ZO-1 immunoreactivity, no changes were observed in retinal wholemounts from diabetic animals (Fig. 5c). No differences were detected in the protein levels of tight junction proteins in retinal homogenates by Western blot (Fig. 5e–g). Despite subtle changes detected in the immunoreactivity of tight junction proteins in retinal wholemounts from diabetic animals, no vascular leakage was observed, as assessed by Evans Blue assay (Fig. 5d).

### Type 2 diabetes does not induce nitrosative stress and glial reactivity in the retina

Diabetic retinas were assessed for nitrosative stress by immunofluorescence in retinal cryosections. Additionally, to evaluate the effects of type 2 diabetes on neuroinflammation, Müller cell and microglia reactivity was also assessed by immunofluorescence in retinal cryosections, along with the assessment of IL-1β and TNF protein levels in retinal homogenates by Western blot and ELISA. In diabetic animals, there were no signs of nitrosative stress, as evidenced by no significant differences in nitrotyrosine immunoreactivity in the retina (Supplementary Figure S3a and S3d), compared with controls. Further, there was no evidence of neuroinflammation, since no changes were found in Iba1^+^ cell density (microglia/macrophages; Supplementary Figure S3b and S3e), and in the morphology and reactivity of Müller cells (Supplementary Figure S3c and S3f), when compared with controls. In addition, the protein content of IL-1β (Supplementary Figure S3g and S3h) and TNF (Supplementary Figure S3i and S3j) remained unaffected under diabetic conditions.

## Discussion

Significant advances in ophthalmic imaging techniques have been made in recent decades, particularly with the introduction of OCT technology, including OCT angiography more recently, but also with significant improvements in more traditional techniques, such as ultra-widefield retinal photography and ultra-widefield fluorescein angiography. Despite remarkable progress, DR is regarded as a “silent” disease because it is usually diagnosed many years after diabetes onset, at disease stages when significant biological changes have already occurred in the retina. Indeed, molecular and cellular changes linked to neurovascular and physiological dysfunction are known to precede the development of the first clinical signs of DR [11–14]. Since the diagnostic methods currently available in clinical practice are unable to detect such early (molecular and cellular) changes, an earlier diagnosis of DR remains a major challenge in diabetes research, and therefore there is an urgent need for novel strategies.

In a previous study by our group, significant changes in retinal texture were observed between diabetic and control animals in several retinal texture metrics, across all retinal layers, with differences also noted over the course of the disease [32]. These changes were concomitant with structural, functional, molecular, and cellular alterations in the retina. In this study, the same methodological approach was applied to HFD/STZ-induced type 2 diabetic animals, which exhibit milder and more gradually progressing DR-related retinal alterations. The current study aimed to evaluate potential retinal texture changes (very) early after disease onset and assess whether these changes were concomitant with retinal alterations typically linked to the pathophysiology of DR, including structural (layer thickness) and functional (ERG) changes, as well as molecular, cellular, and vascular alterations.

Performing an animal study was fundamental because it enabled us to assess retinal texture (very) early after diabetes onset, potentially at stages in which clinical resources currently available in clinical practice cannot detect any retinal biological alterations. Furthermore, animal models enable the assessment of DR-related molecular and cellular parameters in the retina, which cannot be evaluated in vivo in humans. Considering that current diagnostic resources used in the clinical management of DR can readily detect advanced disease stages, it is important to highlight that our texture analysis approach is not particularly relevant for those cases. Moreover, this study does not replace a clinical study, as human data is fundamental for corroborating the current findings and, most importantly, to validate this texture-based approach. Evidence from animal models is crucial to establish a proof of concept and to provide a solid rationale for subsequent clinical research.

While animal models are invaluable for studying diabetes-induced (very) early retinal changes and the underlying molecular and cellular alterations, they do not fully replicate the full spectrum of retinal abnormalities observed in patients with DR. Furthermore, it remains unclear whether the early retinal alterations detected in diabetic animals represent early disease features and progress to long-term retinal damage. It is also uncertain whether these changes are directly related to diabetes or influenced by other factors. Unlike human patients, animal models typically do not receive any treatment targeting diabetes-related metabolic control, which may limit clinical translation. Nonetheless, as noted above, clinical studies remain mandatory to confirm and validate these findings. Given the strong correlation between HbA1c levels and DR [43], another limitation of this study arises from the low HbA1c levels detected in animals treated with HFD and STZ. Nonetheless, it is important to note that HbA1c levels in diabetic animals were statistically different from those in controls. Furthermore, fasting blood glucose levels were markedly elevated in the diabetic group, strongly highlighting the hyperglycaemic condition.

Diabetes induced significant differences in several texture parameters (autocorrelation, cluster prominence, correlation, homogeneity, IMCII, IDN, INN, and sum average), but only in the IPL and IS/OS. While seven of these metrics were changed in all retinal layers in our previous studies using an animal model of type 1 diabetes [32], here, only two retinal layers presented significant differences in retinal texture. This likely represents an earlier disease stage, also considering that molecular and cellular changes in this type 2 diabetes model are less pronounced and progress more slowly compared to the type 1 diabetes model. The IPL is indeed recognised as one of the retinal layers primarily affected in type 2 diabetic patients without clinical signals of DR [44, 45], whereas the same is not true for the IS/OS layer. Although scientific evidence suggests that the outer retina appears to be less affected than the inner retina in the early stages of the disease [46], the presence of subtle texture changes in the IS/OS indicates that changes in this layer are already occurring. This could somehow reflect subtle biological alterations that have so far been undetectable, suggesting that retinal texture may eventually have the potential to help unmask retinal diseases. Nonetheless, the results gathered in this study reveal that our methodology can identify significant retinal texture changes between diabetic and control animals (very) early after diabetes onset.

Although OCT images offer valuable anatomical information about the retina, evaluating retinal thickness is not sufficient for identifying a specific retinal disease, because retinal thickness is known to be impaired in several eye conditions. In this study, early after the STZ administration (after 8 and 12 weeks on HFD, with STZ injection at week 4), subtle but significant thinning of almost all retinal layers (apart from the NFL-GCL) and of the total retina was detected. It is important to note that many animals (44 control and 45 diabetic animals) and an automated segmentation method were used, which strongly increase the reliability of the retinal thickness results. Surprisingly, animals fed with HFD for 4 weeks already presented a significant decrease in retinal thickness compared to controls. Despite no changes in fasting glucose, this suggests that a higher caloric intake affects the retina. In sum, the findings in this study suggest that diabetic retinas develop retinal thinning early in the course of the disease, which can eventually corroborate the early changes observed in the texture metrics.

Clinical research studies with diabetic patients without DR suggest that the most prominent changes in the ERG response occur in the inner retina, most specifically in the OP responses [47–49], which was also corroborated in pre-clinical studies [50–52]. In this study, diabetic animals presented increased latencies in the scotopic OPs, which is in accordance with the previous observations. Additional significant changes were observed in the ERG recordings, including a decrease in OPs amplitude, although they were sporadic and inconsistent. Thus, the results of the current study provide evidence of early and very low-grade retinal dysfunction by showing that diabetic retinas develop impaired retinal function (very) early after diabetes onset.

Numerous studies have proposed that both neurovascular damage and chronic low-grade inflammation in the retina are clearly involved in the pathophysiology of DR [5, 6, 9, 53], and the scientific community working in the field has been very supportive of this hypothesis. As such, in this study, several parameters related to neuronal, glial, and vascular retinal cells were assessed, as well as changes related to neuroinflammation. While no differences were found in neuroinflammatory and glial reactivity markers, minimal vascular-level changes were noted (subtle decrease in tight junction proteins immunoreactivity). However, these changes did not induce any physiologically relevant effect on vascular permeability. Indeed, given that type 2 diabetic animals presented only minor retinal biological changes at the time points assessed in this study, the breakdown of the blood-retinal barrier would not be expected.

In summary, at the time points assessed in this study (after 4, 8, and 12 weeks on HFD), type 2 diabetes induced only minor retinal alterations in structural, functional, molecular, and cellular parameters commonly associated with DR. Nonetheless, these conditions—a state in which the effects of diabetes on the retina were still minimal—are precisely the optimal circumstances for investigating whether retinal texture could provide information about minor retinal changes at (very) early stages of the disease. Such findings may ultimately prove useful and translatable after validation in clinical studies. Although a direct association between changes in retinal texture and a specific biological process in the retina cannot be established, the full spectrum of molecular and cellular alterations in the diabetic retina may trigger structural rearrangements in the tissue. In addition, alterations to retinal composition caused by early HFD/STZ-induced changes may result in changes in the refractive index of the retina, leading to differences in the OCT signal that can be detected by texture analysis. Altogether, these alterations can lead to changes in retinal texture that may then serve as indicators of the underlying pathogenic processes and reflect aspects of the disease’s pathogenesis.

The texture analysis conveys multivariate data on the spatial distribution of image intensity, providing information on subtle structural differences of the distinct retinal layers. As mentioned before, using an animal model of type 1 diabetes, we recently demonstrated that our texture-based methodology is able to spot differences in the retina under diabetic conditions early after diabetes induction [32]. In this study, type 2 diabetic animals displayed even subtler retinal biological changes than those we previously observed in type 1 diabetic rats, and yet our methodology consistently discriminated retinal texture between diabetic and control animals. Therefore, the present study significantly strengthens the promising value of this texture-based approach for the early detection of DR. Importantly, seven texture metrics were consistently altered in both experimental models, revealing the coherence of texture results and lending considerable robustness to our texture-based approach.

## Conclusions

In conclusion, this study strongly supports the potential of texture analysis in providing powerful quantitative information on the retinal status and clearly evidences the ability to identify changes in (very) early stages of the disease, even when very minimal biological changes are occurring in the retina. Further research on this topic, particularly in human subjects, could enable earlier diagnosis of DR, significantly enhancing the management and prognosis of this vision-threatening disease.

## Supplementary Information

Additional file 1.

## Data Availability

All data generated or analysed during this study are included in this published article and its supplementary information files.

## Abbreviations

2D: 2-Dimensional
BSA: Bovine serum albumin
DR: Diabetic retinopathy
IDN: Inverse difference moment normalised
ERG: Electroretinography
ELISA: Enzyme-linked immunosorbent assay
GFAP: Glial fibrillary acidic protein
HbA1c: Glycated haemoglobin
GLCM: Grey-level co-occurrence matrix
HFD: High-fat diet
IMCI: Information measure of correlation I
IMCII: Information measure of correlation II
INL: Inner nuclear layer
IPL: Inner plexiform layer
IS/OS: Inner/outer photoreceptor segments
IL-1β: Interleukin-1 beta
IP: Intraperitoneal
INN: Inverse difference normalised
NFL-GCL: Nerve fibre layer and ganglion cell layer complex
OCT: Optical coherence tomography
OGTT: Oral glucose tolerance test
OP: Oscillatory potential
ONL: Outer nuclear layer
OPL: Outer plexiform layer
PFA: Paraformaldehyde
MVF: Mean-value fundus
RGC: Retinal ganglion cell
SCP: Superficial capillary plexus
SDS-PAGE: Sodium dodecyl sulphate–polyacrylamide gel electrophoresis
SEM: Standard error of the mean
STR: Scotopic threshold response
STZ: Streptozotocin
TNF: Tumour necrosis factor
ZO-1: Zonula occludens-1
