# Supplementary material for: Early retinal changes in type 2 diabetes detected by texture-based OCT analysis: potential approach for subclinical diabetic retinopathy diagnosis
Source: Eye Vis (Lond). 2025 Sep 3;12:36. doi: 10.1186/s40662-025-00451-3 (PMC12406546; doi:10.1186/s40662-025-00451-3)
Supplement: Supplementary file 1 — Additional file 1. [file 40662_2025_451_MOESM1_ESM.docx]

**Supplementary Tables:**

Supplementary Table S1. Textural parameters extracted from OCT retinal images.

| **Inner plexiform layer** GLCM-based textural parameters | Control group (mean ± SEM) | T2D group (mean ± SEM) | Control group (mean ± SEM) | T2D group (mean ± SEM) | Sig. (ANCOVA test) (*P* value) | Control group (mean ± SEM) | T2D group (mean ± SEM) | Sig. (ANCOVA test) (*P* value) | Control group (mean ± SEM) | T2D group (mean ± SEM) | Sig. (ANCOVA test) (*P* value) |
| --- | --- | --- | --- | --- | --- | --- | --- | --- | --- | --- | --- |
|  | Week 0 | | Week 4 | | | Week 8 | | | Week 12 | | |
| Autocorrelation | 74.9152 ± 0.2387 | 74.2018 ± 0.2336 | 74.7200 ± 0.2628 | 73.9229 ± 0.2698 | 0.220 | 74.3450 ± 0.2314 | 73.0900 ± 0.4106 | 0.040* | 74.2191 ± 0.2129 | 73.1176 ± 0.3357 | 0.011* |
| Cluster prominence | 1384.0364 ± 10.6740 | 1376.8809 ± 9.9257 | 1409.0130 ± 8.8584 | 1421.3720 ± 9.8283 | 0.282 | 1405.2873 ± 8.9801 | 1437.7893 ± 12.8245 | 0.027* | 1385.9445 ± 9.1788 | 1416.3676 ± 10.5440 | 0.022* |
| Correlation | 0.4098 ± 0.0053 | 0.4068 ± 0.0054 | 0.4217 ± 0.0045 | 0.4290 ± 0.0052 | 0.243 | 0.4231 ± 0.0044 | 0.4374 ± 0.0063 | 0.050* | 0.4130 ± 0.0043 | 0.4270 ± 0.0054 | 0.024* |
| Homogeneity | 0.4161 ± 0.0009 | 0.4157 ± 0.0011 | 0.4184 ± 0.0009 | 0.4198 ± 0.0011 | 0.266 | 0.4192 ± 0.0009 | 0.4230 ± 0.0015 | 0.026* | 0.4183 ± 0.0010 | 0.4211 ± 0.0012 | 0.049* |
| Information measure of correlation II | 0.6943 ± 0.0022 | 0.6931 ± 0.0022 | 0.7002 ± 0.0019 | 0.7027 ± 0.0023 | 0.255 | 0.7002 ± 0.0019 | 0.7073 ± 0.0027 | 0.026* | 0.6962 ± 0.0020 | 0.7021 ± 0.0025 | 0.037* |
| Inverse difference moment normalised | 0.9680 ± 0.0003 | 0.9680 ± 0.0003 | 0.9687 ± 0.0002 | 0.9691 ± 0.0003 | 0.240 | 0.9689 ± 0.0002 | 0.9696 ± 0.0003 | 0.062 | 0.9684 ± 0.0002 | 0.9691 ± 0.0003 | 0.025* |
| Inverse difference normalised | 0.8800 ± 0.0005 | 0.8798 ± 0.0005 | 0.8811 ± 0.0004 | 0.8818 ± 0.0005 | 0.249 | 0.8815 ± 0.0004 | 0.8830 ± 0.0006 | 0.047* | 0.8808 ± 0.0004 | 0.8821 ± 0.0005 | 0.029* |
| Sum average | 16.9236 ± 0.0269 | 16.8416 ± 0.0250 | 16.8850 ± 0.0297 | 16.7853 ± 0.0298 | 0.158 | 16.8407 ± 0.0256 | 16.6738 ± 0.0518 | 0.026* | 16.8327 ± 0.0241 | 16.6871 ± 0.0395 | 0.003* |
|  |  |  |  |  |  |  |  |  |  |  |  |
| **Inner/outer photoreceptors segments**  GLCM-based textural parameters | Control group (mean ± SEM) | T2D group (mean ± SEM) | Control group (mean ± SEM) | T2D group (mean ± SEM) | Sig. (ANCOVA test) (*P* value) | Control group (mean ± SEM) | T2D group (mean ± SEM) | Sig. (ANCOVA test) (*P* value) | Control group (mean ± SEM) | T2D group (mean ± SEM) | Sig. (ANCOVA test) (*P* value) |
|  | Week 0 | | Week 4 | | | Week 8 | | | Week 12 | | |
| Autocorrelation | 73.4193 ± 0.4043 | 72.3600 ± 0.4178 | 75.1768 ± 0.2684 | 74.1756 ± 0.5853 | 0.556 | 75.6773 ± 0.2445 | 71.8802 ± 0.6342 | 0.001* | 76.0441 ± 0.3273 | 73.4051 ± 0.5982 | 0.001* |
| Cluster prominence | 1633.2986 ± 23.9146 | 1594.0251 ± 22.0279 | 1737.0807 ± 25.3218 | 1703.9104 ± 28.3530 | 0.765 | 1779.0993 ± 31.8619 | 1665.2296 ± 33.9115 | 0.038* | 1754.0925 ± 28.0271 | 1645.7351 ± 34.9789 | 0.043* |
| Correlation | 0.4318 ± 0.0073 | 0.4181 ± 0.0066 | 0.4623 ± 0.0055 | 0.4498 ± 0.0087 | 0.430 | 0.4670 ± 0.0056 | 0.4325 ± 0.0086 | 0.003* | 0.4570 ± 0.0054 | 0.4291 ± 0.0082 | 0.016* |
| Homogeneity | 0.4198 ± 0.0015 | 0.4171 ± 0.0013 | 0.4263 ± 0.0012 | 0.4234 ± 0.0017 | 0.352 | 0.4273 ± 0.0013 | 0.4201 ± 0.0017 | 0.003* | 0.4260 ± 0.0013 | 0.4197 ± 0.0016 | 0.008* |
| Information measure of correlation II | 0.7075 ± 0.0030 | 0.7025 ± 0.0027 | 0.7204 ± 0.0024 | 0.7167 ± 0.0036 | 0.636 | 0.7228 ± 0.0025 | 0.7094 ± 0.0036 | 0.006* | 0.7189 ± 0.0025 | 0.7075 ± 0.0036 | 0.023* |
| Inverse difference moment normalised | 0.9686 ± 0.0004 | 0.9679 ± 0.0003 | 0.9702 ± 0.0003 | 0.9694 ± 0.0004 | 0.273 | 0.9704 ± 0.0003 | 0.9684 ± 0.0004 | 0.001* | 0.9699 ± 0.0003 | 0.9684 ± 0.0004 | 0.006* |
| Inverse difference normalised | 0.8814 ± 0.0007 | 0.8800 ± 0.0006 | 0.8843 ± 0.0005 | 0.8829 ± 0.0008 | 0.307 | 0.8847 ± 0.0005 | 0.8811 ± 0.0008 | 0.001* | 0.8839 ± 0.0005 | 0.8810 ± 0.0007 | 0.006* |
| Sum average | 16.6691 ± 0.0445 | 16.5571 ± 0.0467 | 16.8389 ± 0.0278 | 16.7304 ± 0.0621 | 0.508 | 16.8868 ± 0.0248 | 16.4720 ± 0.0683 | 0.001* | 16.9420 ± 0.0352 | 16.6644 ± 0.0635 | 0.001* |

GLCM = grey-level co-occurrence matrix; HFD = high-fat diet; IPL = inner plexiform layer; IS/OS = inner/outer photoreceptor segments; OCT = optical coherence tomography; STZ = streptozotocin; T2D = type 2 diabetes; SEM = standard error of the mean.

Mean ± SEM of GLCM-based textural parameters extracted from OCT retinal images of diabetic Wistar Han rats (induced by a 12-week HFD with STZ injection at week 4; 35 mg/kg, IP; T2D group; *N* = 45) and age-matched controls (Control group; *N* = 44) of the retinal layers IPL and IS/OS. Statistical analysis was performed using the ANCOVA test, considering the baseline values (week 0) as a quantitative covariate. **P* ≤ 0.05, versus the age-matched Control group.

**Supplementary Figures:**

**
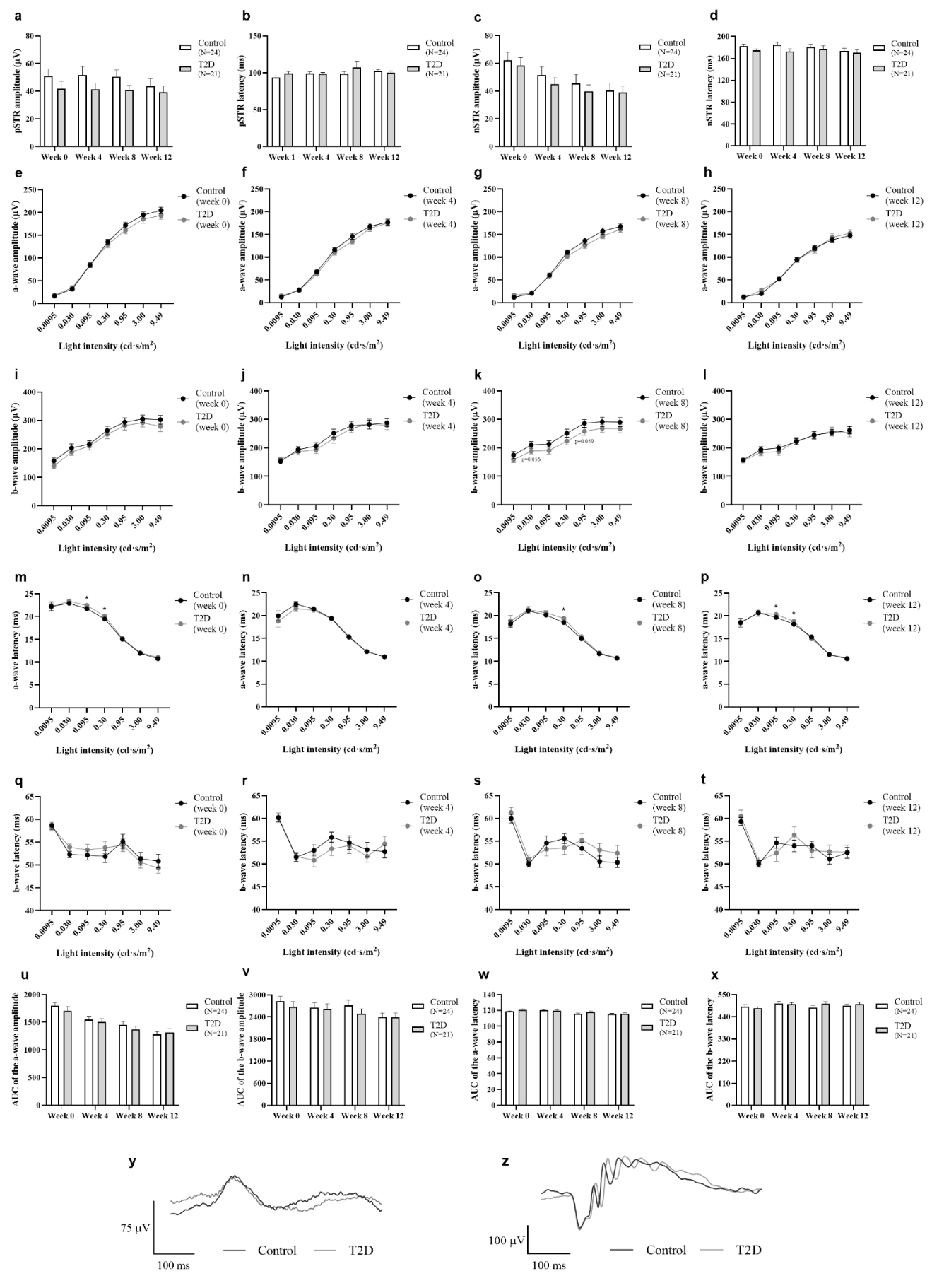
Supplementary Figure S1.** Diabetes does not exert pronounced effects on scotopic ERG. Diabetic Wistar Han rats (induced by a 12-week HFD with STZ injection at week 4; 35 mg/kg, IP; T2D group; *N* = 21) presented only subtle changes on scotopic ERG compared to age-matched control rats (Control group; *N* = 24). The positive scotopic threshold response (pSTR) and the negative scotopic threshold response (nSTR) (**a**, **c**) amplitudes and (**b**, **d**) latencies in response to a 0.000095 cd·s/m^2^ light stimulus. Scotopic a-wave and b-wave amplitudes in response to 0.0095–9.49 cd·s/m^2^ light stimuli recorded at (**e**, **i**) week 0, and after (**f**, **j**) 4, (**g**, **k**) 8, and **(h**, **l)** 12 weeks on HFD. Scotopic a-wave and b-wave latencies in response to 0.0095–9.45 cd·s/m^2^ light stimuli recorded at (**m**, **q**) week 0, and after (**n**, **r**) 4, (**o**, **s**) 8, and **(p**, **t)** 12 weeks on HFD. Area under the curve (AUC) of scotopic a-wave and b-wave **(u**, **v)** amplitudes and (**w**, **x**) latencies. Representative traces of (**y**) STR and (**z**) scotopic waves of control and diabetic animals recorded after 12 weeks on HFD. Results are presented as mean ± SEM. Statistical analysis was performed using the ANCOVA test, considering the baseline values (week 0) as a quantitative covariate. **P* ≤ 0.05, versus the age-matched Control group. ERG, electroretinography; HFD, high-fat diet; STZ, streptozotocin; T2D, type 2 diabetes; STR, scotopic threshold response; SEM, standard error of the mean

**Supplementary Figure S2.** Diabetes has no relevant effects on the photopic ERG. Diabetic Wistar Han rats (induced by a 12-week HFD with STZ injection at week 4; 35 mg/kg, IP; T2D group; *N* = 21) presented just a few changes on photopic ERG compared to age-matched control rats (Control group; *N* = 24). Photopic b-wave (**a**) amplitude and (**b**) latency in response to the higher light intensity stimulus (9.49 cd·s/m^2^). Flicker **(c**, **d**, **e)** base wave, (**f**, **g**, **h**) 1st, and (**i**, **j**, **k**) 2nd harmonic amplitudes recorded in response to 0.95, 3, and 9.49 cd·s/m^2^ light stimuli, respectively. Representative traces of (**L**) photopic wave and (**m**) flicker responses of control and diabetic animals recorded after 12 weeks on HFD. Results are presented as mean ± SEM. Statistical analysis was performed using the ANCOVA test, considering the baseline values (week 0) as a quantitative covariate. **P* ≤ 0.05, ***P* < 0.01, versus the age-matched Control group. ERG, electroretinography; HFD, high-fat diet; STZ, streptozotocin; T2D, type 2 diabetes; SEM, standard error of the mean


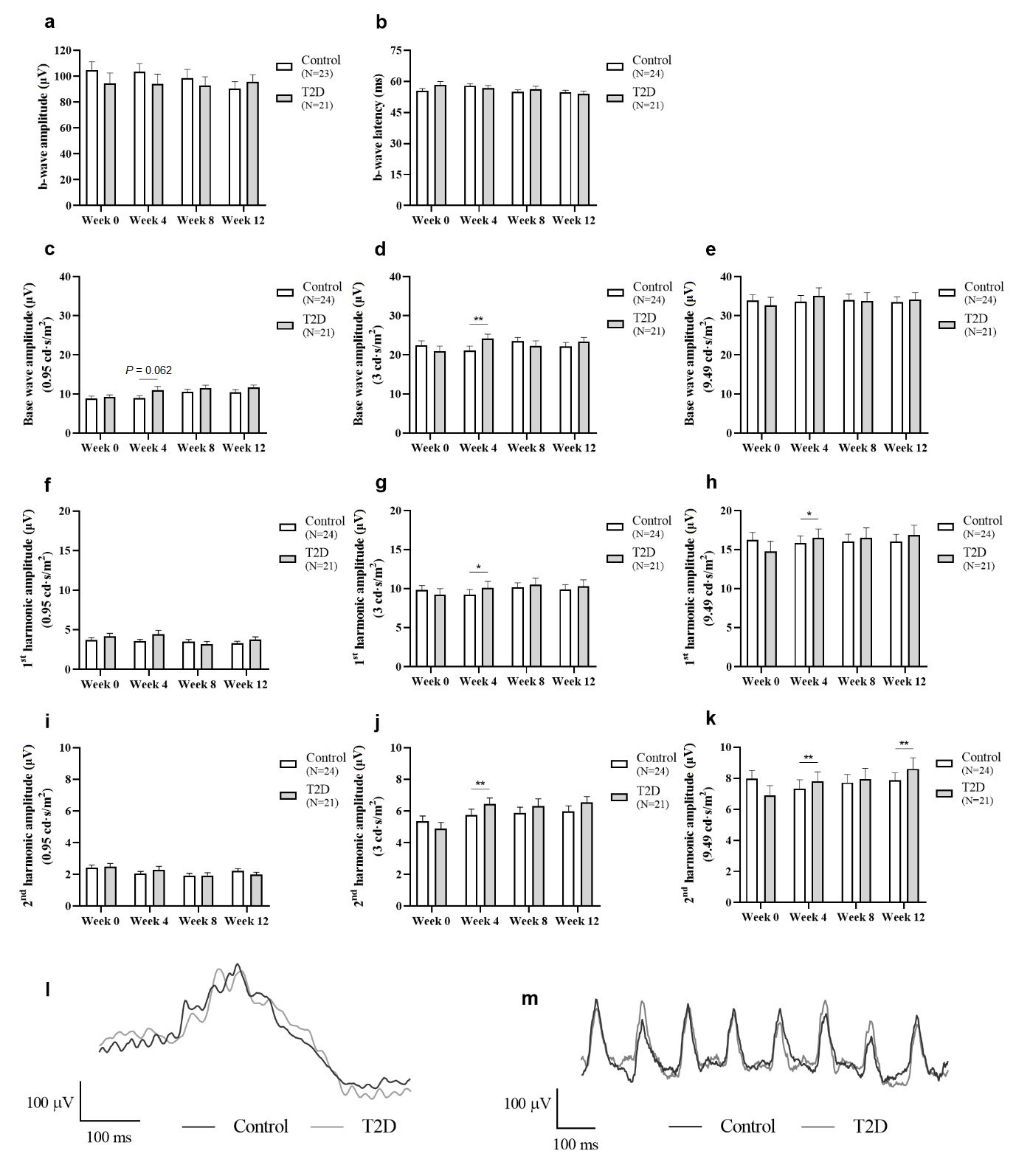


**
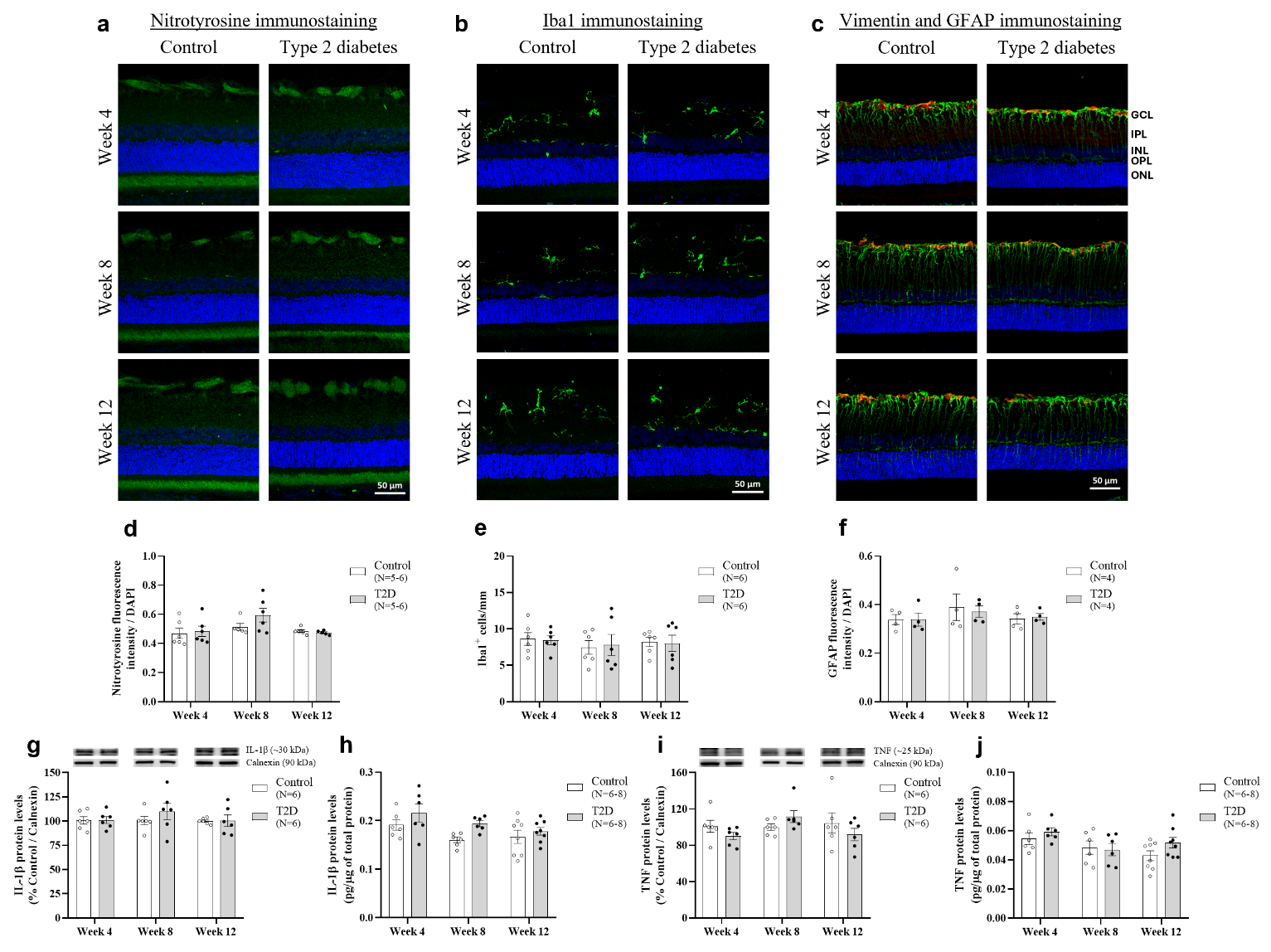
Supplementary Figure S3**. Type 2 diabetes does not trigger nitrosative stress, glial reactivity, and neuroinflammation in the retina. Retinas from diabetic Wistar Han rats (induced by a 12-week HFD with STZ injection at week 4; 35 mg/kg, IP; T2D group) presented no differences in nitrosative immunoreactivity as well as in the morphology and reactivity of microglial and Müller cells, compared to age-matched controls (Control group). The protein levels of pro-inflammatory cytokines also remained unchanged. Representative images of retinal cryosections immunoassayed for (**a**) nitrotyrosine, (**b**) Iba1, and (**c**) vimentin and GFAP. The quantification of nitrotyrosine immunostaining, Iba1^+^ cells (microglia), and GFAP immunolabeling are presented below (**d**, **e**, and **f**, respectively). (**g**) IL-1β and (**i**) TNF protein levels were assessed by Western blot, normalised to the loading control (calnexin), and expressed as a percentage of the respective control. Representative images of protein immunoreactive bands are presented above the graphs, with the respective loading control (calnexin). (**h**) IL-1β and (**j**) TNF protein levels assessed by ELISA. Data are presented as mean ± SEM. Statistical analysis was performed using the Student’s *t*-test, or the Mann-Whitney test, if data were not normally distributed. HFD, high-fat diet; STZ, streptozotocin; T2D, type 2 diabetes; GFAP, glial fibrillary acidic protein; TNF, tumour necrosis factor; IL-1β, interleukin-1 beta; SEM, standard error of the mean
